# Clinical Lectures in the Manchester Royal Infirmary

**Published:** 1835-01-01

**Authors:** 


*Clinical Lectures in the Manchester Royal Infirmary.* By Edward Carbutt, M.D.—London, 1834. 8vo. pp. 407.

The author of this work appears to be a diligent physician, who, having ample opportunities of improving in the practice of physic, does not suffer them to pass by, and will one day, we trust, become an eminent master of the art. At present, Dr. Carbutt is haunted by the fear of gastro-enteritis to a degree which is perfectly astounding; for this Gallic generalization comprehends, with him, not merely dyspepsia, and fever, and other such diseases, where this inflammation of the digestive tube might make out a decent claim to prior possession, but he thinks gastro-enteritis entitled to lord it over hydrophobia, delirium tremens, and diabetes. Nay, more; phthisis depends on the same unsuspected cause.

"Gastro-enteritis accompanies or fatally terminates some diseases; as is seen in patients who die of phthisis, who are almost always carried off by a colliquative diarrhœa, and who, upon being examined after death, are found to have had the mucous membrane of the alimentary canal inflamed and ulcerated. I am inclined to think that, in these cases, the gastro-enteritis is the primary disease, and that it gives rise to the phthisis somewhat in this manner. You know that gastro-enteritis is a very insidious, obscure, and lurking disease. Well, we will suppose a patient with delicate and irritable lungs to labour under an unsuspected gastro-enteritis for several months; this produces cough in the manner explained already at page 15. This cough irritates the lungs, produces increased expectoration, inflammation, tubercles, and abscesses; in short, phthisis. This disease attracts the attention; the gastro-enteritis is quite overlooked until the colliquative diarrhœa comes on, and even then the patient is said to have died of phthisis with diarrhœa, the existence of gastro-enteritis being never suspected from the beginning to the end. We had a patient of the name of Peacock, who died in this manner in this house, two years ago; upon examining his lungs, after death, we found comparatively little disease in them, although most people would have said the man had died of phthisis: but his stomach, duodenum, ileum, colon, and rectum, were both inflamed and ulcerated. The ulcers were so numerous, that the best mode of giving you an idea of them, is to ask you to conceive a vertical section of a bunch of grapes. Our worthy house-apothecary, who had conceived the disease to be pure phthisis, told me, after the inspection, that he had never been more surprised in his life. The man, in fact, died of gastro-entero-colitis; for which disease I had treated him." (P. 24.)

We are surprised at the surprise of the worthy house-apothecary, and doubly surprised at our author's saying that in such cases the existence of gastro-enteritis is never suspected from beginning to end. The fact that phthisis, in its last stage, is very often complicated with ulceration of the bowels, is now familiar to every one: we believe that it was first pointed out by Dr. Thomas Young, in his Treatise on Consumption, who mentions that it had been observed by Dr. Nevinson. A little further on, our author says,

"Every man who is intoxicated has, at the time, and for a number of hours afterwards, a gastro-enteritis, which is shown by his great thirst, burning pain at his stomach, nausea and vomiting, headach, pain and weariness in his limbs, his loss of appetite, and the lowness of his spirits. Give him absolutely nothing to eat, and nothing to drink except cold water, and let him lie in bed, and he speedily recovers. Hence we have a hint for the treatment of gastro-enteritis. Abstinence and repose." (P. 26.)

True, if the case be one of real active inflammation; but this is comparatively rare. In a very common class of dyspeptic cases, where there is a regurgitation of bile into the mouth, we may take a different hint for the treatment from our author's illustration of a drunken man: we may give an emetic.

We extract the following case, partly because it is very short, and partly to show the zeal with which our author presses everything into the gastro-enteritic service.

"Gastro-enteritis chronica; form, hysteria. 1st July, 1833. Mary Booth, aged twenty-five years, unmarried, works in a cotton-factory. Has been ill two years. Says she has never felt well since she bathed at Liverpool. She now complains of a sinking, as if she were going to faint. This is generally relieved by eating. Her appetite is rather increased than diminished Tongue rough, and slightly red at the tip and edges. Is much troubled with wind. Bowels regular. Pulse weak. Has occasionally pain in the back, hips, thighs, legs, and calves. No pain in the epigastrium on pressure. Has been troubled with unpleasant dreams. Catamenia regular.—Common poultices to the epigastrium and belly. To lie in bed. Rice diet, biscuit, and barley-water. Castor-oil occasionally.

"2d. Is much better. Slept well. No sinking.

"3d. Tongue clean. Pulse stronger.

"4th. Has been troubled with globus hystericus.

"5th. Much better.

"6th. No complaint.

"13th. Had an hysteric fit this morning, which subsided, upon her being threatened with the affusion of cold water.

"14th. Has no unhealthy symptom. A bucket of cold water is kept in readiness, but she has had no recurrence of the fit.

"15th. Discharged cured.

"*Observations.* Much will not be required to be said on this case. I would, however, just call your attention to the fact of the great efficacy of the fear of an effusion of cold water, in removing or preventing the hysteric paroxysm; and I can assure you, from much experience, that if the fear be so efficacious, the real affusion is still more so. It is, in fact, an infallible remedy; the principal objection against it being that females are exceedingly averse to it, and do not like to be cured of hysterics in this manner. I have never known but two females of the better class of society who took this remedy in really good part. You must therefore be guided by circumstances, and cure your patient either by this ready, infallible, and radical remedy, or go on for weeks, with aloes, assafœtida, valerian, castor, and ether, accordingly as you judge your patient and her friends will be the better pleased." (P. 54.)

Surely there were no marks of inflammation here, though we are aware that "chronic" inflammation is a drag-net that will hold most fish. Poor Mary Booth was an overworked factory-girl, sinking under the slavery of a cotton-mill, and a fortnight's repose in the Infirmary did wonders for her. The cold affusion is an admirable remedy in hysteria, and our author's observations upon it show the practised physician.

As it is well to have a choice of remedies, it is useful to know that hysterical patients usually have a tender spot in the spine; and, if this be blistered, they are much relieved: for this fact we believe that pathology is indebted to Dr. Griffin.

The next case is also called one of chronic gastro-enteritis; and the following is the first day's report and prescriptions.

"Elizabeth Schofield, aged thirty, married, and a weaver, has been ill eighteen months. Her complaint first came on with pain in the stomach, accompanied by pains in the back, shoulders, arms, thighs, and legs, rendering her unable to stand or follow her employment. Headach. Sleep disturbed by alarming dreams. Tongue streaked with red and white. Great thirst, calling for frequent draughts of cold water. Unpleasant taste in the mouth. Bad appetite. Bowels costive. Much wind. Skin moist. Pulse 110, weak. No catamenia for seven weeks.—Ten leeches to the epigastrium. Poultices afterwards. Castor-oil occasionally. To lie in bed; and take rice-diet, biscuit, and barley-water." (P. 56.)

In cases of this kind we should recommend an emetic on the first day, and afterwards something of this kind:

℞. Aq. Menth. Pip. ℥viij.

Rhei pulv. ʒss.

Zing. pulv. ℈j.

M. sumat coch. iij. majora ter in die.

This prescription is of course intended for institutions where the most rigid economy is a matter of dire necessity: under more favourable circumstances, it would be neater to substitute the Inf. Rhei and the Tr. Zingib. The mixture, however, as given above, has been prescribed by us innumerable times, with almost undeviating success. On the twenty-fifth day of this case, we find that the patient felt very sick, and vomited a small quantity of bilious matter. Was not this an instance of Nature's asking, in the plainest terms, for Ipec. rad. pulv. ℈j.?

There is in the book a case of ague treated by the endermic method. A blister was applied to the abdomen, and the vesicated surface being daily dressed with ten grains of sulphate of quinine, a cure was effected in five days.

In his observations on a case of turgescence of the spleen, Dr. Carbutt observes,

"If this man had not been affected with gastro-enteritis, I think he might have been cured by a liberal use either of the real Cheltenham water, or of an imitation of it. We come near enough in the imitation, if we dissolve two grains of sulphate of iron, four drachms of sulphate of magnesia, and four drachms of sulphate of soda, in a pint of water, and add twenty or thirty minims of tincture of iodine; this quantity to be taken every twenty-four hours, using reasonable exercise in the meantime. However, I was obliged to content myself with giving him tincture of iodine and laudanum, for his principal complaint, and the hydrargyrum cum cretâ with opiate confection for his diarrhœa, and some affection of the liver, under which he evidently laboured.

"The patient was very little benefited; and, if I must speak my candid opinion, I am inclined to think that his complaints will never be removed." (P. 198.)

Now it seems to us that this man might have ingurgitated his pint of imitation-Cheltenham water per day with the greatest safety, in spite of his gastro-enteritis, especially if our author had made a closer approximation to the real one; for it appears, by the following quotation, which he gives soon afterwards, that iodine exists in Cheltenham water in the proportion of a grain, not in one pint, but in ten or more gallons.

"'It seems not improbable,' says Dr. Daubeny, Professor of Chemistry, at Oxford, 'that very minute portions of certain principles may act upon the system with an energy commensurate, not to their own quantity, but to the change their presence occasions in the properties of the more inert ingredients that accompany them. In this manner we may explain the powerfully tonic effects of certain springs containing a very minute impregnation of iron; the cures effected by waters, such as those of Loueche or Gastein, which appear to approach as nearly as possible to absolute purity; and the efficacy in glandular disorders attributed to certain others, in which a minute proportion of iodine or bromine has been detected. In a memoir read before the Royal Society, on the saline and purgative springs of this country, in which I stated the proportions of iodine and bromine present in each, I expressed myself as being sceptical with regard to any medicinal agency that could be exerted by so small a quantity as one grain of iodine diffused through ten gallons of water, the largest quantity in which I had ever detected it. The considerations above stated now induce me to attach more importance to the circumstance of its presence, for it is just as possible, *à priori*, that this quantity of iodine should infuse new properties into the salts which accompany it, and cause them to act in a different manner upon the system, as that less than a millionth part of potassium should create so entire a change in the relations of a mass of mercury to electricity. Whether the waters of Cheltenham or Leamington affect the constitution differently from solutions of glauber-salt of similar strength, must be decided by the experience of those on the spot; but, granting this to be the case, and there is not wanting testimony in favour of such an opinion, the discovery of these new principles in several of them may serve to explain their superiority.' (P. 202.)

Our author's lecture on Dropsy, from p. 244-274, is very instructive; and so are the cases which follow it. He says, that when a patient has taken as much mercury as he can well bear, a small dose of aloes should be frequently repeated through the day, and will have the same effect in restoring the healthy action of the liver. Thus, at p. 276, Margaret Kearney is ordered to take two grains of Pil. Aloës cum Myrrhâ every hour whilst waking; and at p. 310, Sarah Holland is ordered to take three grains every hour, with the same limitation.

The work terminates with observations on Diabetes, and a number of cases; and, as they are not very common, we will give a brief abstract of each.

*Cases of Diabetes insipidus.*

John Wright, a labourer, æt. sixty-four, was admitted the 19th of June, 1833. The first day his drink amounted to five pints, his urine to nine; its specific gravity being 1006. The quantity of urine was brought down to two pints; but he died on the 3d of July, of ulceration of the stomach and intestines. The kidneys were mottled and granulated.

Hannah Wardle, a sempstress, æt. forty, was admitted August 12th, 1833. Her drink on the following day amounted to twenty-eight pints, and her urine to thirty; on the 16th she drank seventy pints, and passed sixty. She was discharged, relieved, December 9th, when her drink amounted to ten pints, and her urine to nine.

John Ogden, a weaver, æt. sixty, was admitted on the 16th of December, 1833. At first, he passed eight or nine pints of urine daily, of the specific gravity of 1009. He was going on very well, and the quantity was reduced to seven pints; when he was carried off by a paralytic attack, a little more than a fortnight after admission. He had been subject to fits, probably epileptic, for some months.

Richard Rice, a boy, æt. seventeen, working in a cotton-factory, was admitted on the 11th March, 1834. The quantity of urine voided was then nine pints, of the specific gravity 1008. The urine was very soon reduced to five pints a day, of the specific gravity 1005. Rice had been in the house a short time before, labouring under the same disease, and had been discharged relieved.

Dr. Carbutt observes, "This is a case of successful treatment of the insipid diabetes. The patient had the sulphur bath and the vapour bath, the compound powder of ipecacuan, leeches to the stomach, and castor-oil when required. When he was finally discharged, his urine contained a plentiful quantity of urea." (P. 398.)

The remaining cases are all of Diabetes mellitus: we shall throw them into the form of a table, to save room.

| Patient's Name. | Age. | Pints of Urine passed when admitted. | Result. |
|---|---|---|---|
| Wm. Grimshaw | 37 | 21 | Much relieved in three months. |
| Francis Isdale | 51 | 20 | Cured in two months. |
| Thomas Blackwell | 17 | 15 | Relieved in four months. |
| Hugh Capstick | 39 | 8 | Much relieved in a month. |
| Henrietta Wilson | 39 | 12 | Died in a week. |
| Hannah Lees | 38 | 15 | Slightly relieved in seven weeks. |
| Mary Kay | 35 | 25 | Relieved in three months. |
| Ann O'Neale | 26 | 17 | Died in three months. |
| Joseph Taylor | 39 | 14 | Much relieved in two months. |
| Henry Bentley | 6 | 27 | |

It is not stated whether this last patient died or not; but Dr. Carbutt mentions his want of success in treating the case, and attributes it to the child's delicacy, which contraindicated bleeding and leeching. His weight however increased, during his three months' stay in the Infirmary, from thirty-five and a half to thirty-eight and a half pounds, while the quantity of urine passed in twenty-four hours decreased from twenty-seven to seventeen pints. Our author bleeds and leeches in this disease, but prescribes opium and the warm bath, like other people.

This book is the work of a sensible, painstaking, and very candid practitioner: we recommend it to our readers, especially our younger ones.

